# Temperament Assessment Algorithm in Dogs

**DOI:** 10.3390/ani12050634

**Published:** 2022-03-02

**Authors:** Mirosław Karpiński, Justyna Wojtaś, Aleksandra Garbiec

**Affiliations:** Department of Animal Ethology and Wildlife Management, University of Life Sciences in Lublin, Akademicka 13, 20-920 Lublin, Poland; miroslaw.karpinski@up.lublin.pl (M.K.); justyna.wojtas@up.lublin.pl (J.W.)

**Keywords:** dog, temperament, personality, extrovert, introvert

## Abstract

**Simple Summary:**

The assessment of the personality and temperament of an animal is becoming more popular and appreciated not only by the keepers of working dogs, but also by the owners of companion dogs. The aim of our work was to create a short questionnaire in the form of a table for animal keepers with 24 specific temperament traits listed. Two veterinarians and forty-six animal behaviorists (owners of the observed dogs) participated in the study by observing the behavior of dogs in their natural environment. On the basis of the selected features, the temperament of the dogs was determined and assigned to one of two groups: introverts or extroverts.

**Abstract:**

The aim of this study was to evaluate the temperament of dogs on the basis of behavioral observations, with emphasis on 24 selected traits and behaviors. From the observations, the temperament of the dogs was determined and the animals were assigned to one of two personality groups: introvert or extrovert. The study involved 46 dogs. The agglomeration method, Pearson’s 1-*r* distance, and Ward’s binding method were used. As shown by the statistical analysis, 18 dogs (39%) were assessed as introverts and 28 dogs (61%) exhibited extrovert traits. To construct a model for the assessment of canine temperament using the identified traits, logistic regression was performed with the independent variables, number of extrovert traits (ETs) and introvert traits (ITs), and a dichotomous dependent variable (1 = extrovert, 0 = introvert), reflecting the assessment of the temperament of the dog based on the observations and results of the original questionnaire.

## 1. Introduction

Personality and temperament are terms used in psychology to describe different ways of feeling and behaving. Temperament is an essential part of personality [1]. There are two basic terms in the ethological literature, i.e., temperament and personality. Personality is understood as a set of psychological traits that determine the reaction of an animal to a given situation [2]. Temperament, in turn, is understood as an innate tendency to display certain traits [1]. Temperament is defined as the biological and instinctive part of personality. In fact, this part of personality always shows first. The first discoveries in this field were made by Ivan Pavlov, who studied dog conditioning to associate a specific sequence of events with stimuli [3]. Since it is primarily determined by innate physiological mechanisms, changes in temperament are induced by puberty, aging, and environmental factors. Approximately 40% of temperament traits have a genetic (hereditary) background, whereas 50–60% of these traits are determined environmentally. Hence, the same dog examined at the age of 1 year and again at the age of 10 years may turn out to have the same temperament traits, although manifested differently. Temperament cannot be evaluated as being either good or bad; it is relatively constant, but manifests itself in variable behaviors [4]. It influences the development of personality and its traits. A very strong impact of temperament has been detected in puppies. At the stage of adaptation to the environment during further development, its influence on behavior weakens and the impact of the experience gained becomes important.

The most common method for assessment of human personality is the Eysenck Personality Questionnaire-Revised [5]. In accordance with the personality theory proposed by Costa and McCrae [6], the assessment is based on one of the five factors of the personality model, i.e., extroversion. The so-called “big five” personality traits should be regarded as a tendency to behave in a certain way in a situation that is conducive to its expression. For example, a dog may be both an extrovert, with readiness to retrieve and jump in a play-provoking situation, and an introvert, guarding resources and its individual comfort zone in the household. This does not mean a change in the trait; the change lies in the situation (expression) in which the dog exhibits introverted behavior despite its extroversion, or vice versa. There is no contradiction in this claim. In the DPQ (dog personality questionnaire) personality test battery, the authors assessed dog temperament traits (e.g., self-confidence, aggression, excitability, self-control, distance, submissiveness, and assertiveness) in different situations. These included walking on a leash with a familiar person/stranger, a friendly/threatening situation, being stroked by a familiar person/stranger, being grabbed by the neck, and contact with a novel object, etc. [7].

Observations of the behavior of a given animal allow the assignment of a specific personality trait, as in the present study. Dog personality traits such as boldness, exploration, aggression, activity, and social tendency are assessed most frequently [8]. Although animals cannot answer the study questions, researchers use a questionnaire method to evaluate the temperament traits of dogs based on responses obtained from their owners [9]. Since keepers know their pets best and observe them throughout their lives in a variety of situations, it is believed that their answers to the questions can be considered a reliable source of information about the personality of the dog, as demonstrated by Gosling [10]. The most popular questionnaire for the assessment of canine personality is Serpell’s Canine Behavioral Assessment and Research Questionnaire (C-BARQ), which contains 101 questions [11]. Considerable agreement of results has been shown between the C-BARQ test and DMA (Dog Mentality Assessment) behavioral test [12]. An increasing number of scientists investigating dog personality traits use questionnaires/surveys addressed to the animal keeper, and these exhibit great efficiency in the determination of personality types.

One of the many theories of personality assessment is Eysenck’s theory, which is based on division into introverted and extroverted personality types. A study conducted by Gosling and John [2] showed similarities in specific personality traits between humans and dogs. However, there is no well-validated, reliable, and effective instrument for dog personality assessment characterized by easy and quick use and a wide range of applications.

The aim of the study presented herein was the development of a shortened questionnaire that would be understandable and simple to an animal owner who is not an animal behaviorist. The questionnaire was created with the involvement of veterinarians (supervisors–originators) and animal behaviorists (owners of the tested dogs). However, it was targeted at all dog owners to help them understand the dog’s type of temperament in a clear and friendly manner.

An additional application value of the developed algorithm is that it can be used during a short visit to the veterinary practice, while admitting the dog to a hotel, or during behavioral consultations. There are many scientific papers reporting that the determination of dog’s temperament takes several days and is a very complex process. The authors of this study aimed to produce a completely different/practical assessment of temperament.

## 2. Materials and Methods

The study involved 46 dogs and their keepers (certified animal behaviorists) whose task was to indicate the presence or absence of the analyzed traits in the dogs in the designed questionnaire (Table 1). The test leaders (two veterinarians) were responsible for guiding the owners to fill in the test correctly. The dogs were observed and evaluated under neutral environmental conditions without stressful or distracting stimuli. All dogs were domestic companion dogs who did not show extreme features, such as excessive aggression or excessive timidity, and extreme expression in an animal with a balanced character was noted during the behavioral observations. They were also clinically healthy and neutered. The authors deliberately did not take into account such parameters as sex, age, breed, or type of maintenance in the subsequent analyses.

The traits shown in the table were selected from many scientific publications [7,12,13,14] in order to choose only typical extroverted and introverted temperamental traits and to minimize the time and resources required for assessment of the temperament of the dogs. Statistical analysis of the results was conducted using the agglomeration method, Pearson’s 1-*r* distance, and Ward’s binding method.

## 3. Results

**Figure 1:**
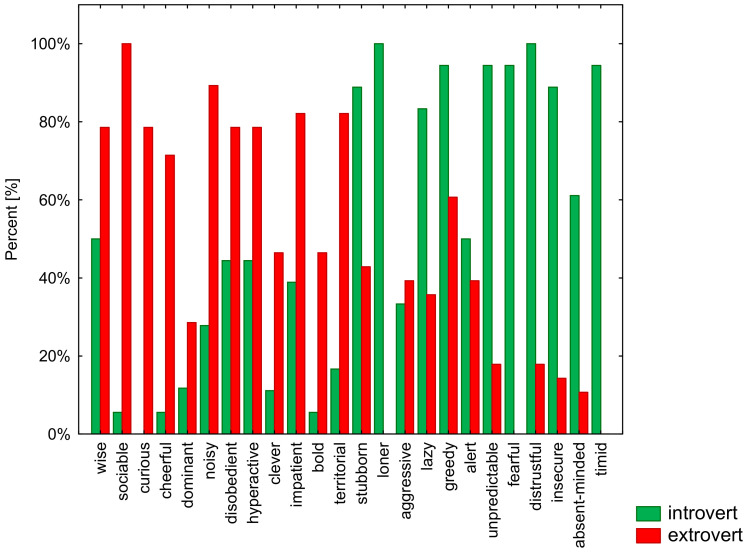
Percentage distribution of the observed traits in both subject groups.

**Figure 2:**
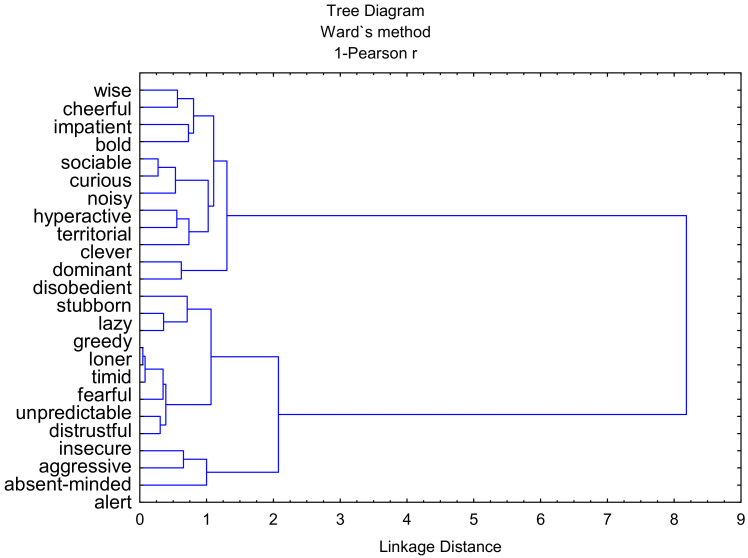
Hierarchical tree chart with two clusters of features.

In the study group, the temperament of 18 dogs (39%) was assessed as introverted, while 28 dogs (61%) exhibited an extroverted temperament (Figure 1 and Table 2). Moreover, two clusters of the analyzed traits visible in the hierarchical tree diagram were detected (Figure 2). Cluster 1 comprised the following traits: wise, cheerful, impatient, bold, sociable, curious, noisy, hyperactive, territorial, clever, dominant, and disobedient. In turn, cluster 2 comprised the following traits: stubborn, lazy, greedy, loner, timid, fearful, unpredictable, distrustful, insecure, aggressive, absent-minded, and alert. Notably, the traits from cluster 1 were assigned to individuals with an extroverted temperament, while the traits from cluster 2 are typical of introverted animals. This study thus confirms the same grouping of temperament traits in dogs.

The number of ETs (in cluster 1) and the number of ITs (in cluster 2) indicated in the questionnaire of each dog were counted. In order to construct a model for the assessment of the temperament of the dogs based on the survey results, a logistic regression was performed with the independent variables ETs and ITs and a dichotomous dependent variable *Y* (1 = extrovert, 0 = introvert), indicating the temperament based on the observations of the behaviorist.

A logistic regression model with parameters presented in the table below (Table 3) was developed:

(1) $$P\;\left(Y=1|ET=x,IT=y\right)=\frac{\exp\left(a_{0}+a_{1}x+a_{2}y\right)}{1+\exp\left(a_{0}+a_{1}x+a_{2}y\right)},$$

where $P\;\left(Y=1|ET=x,IT=y\right)$ is the probability that the dog is extroverted if *ET* = *x* and *IT* = *y*.

**Figure 3:**
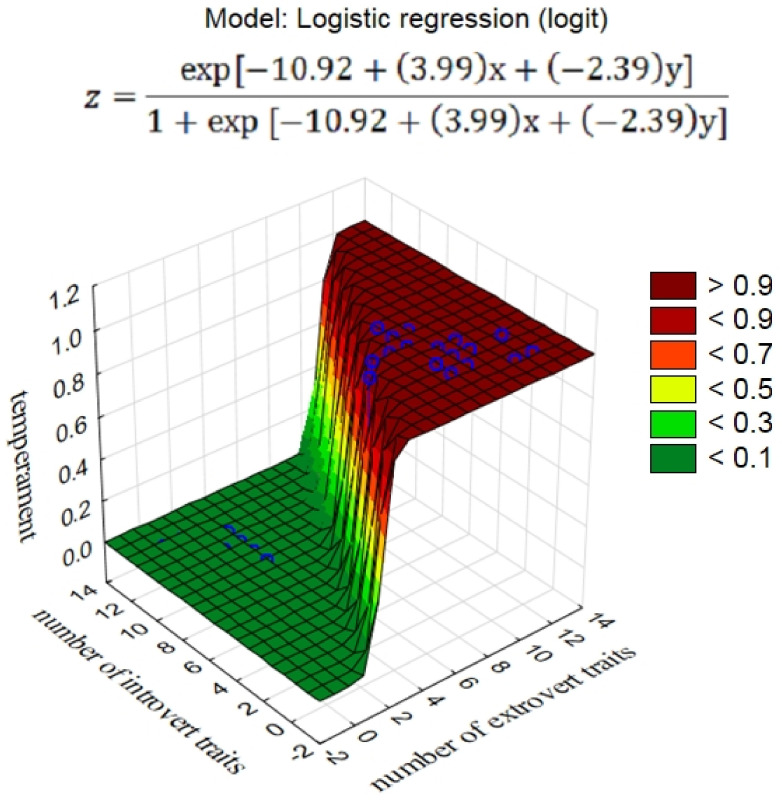
Logistic regression model.

In the logistic regression model, the substitution of the number of extrovert and introvert traits, as well as model parameters, to the right side of the equation for each analyzed dog yielded the value of the probability that the dog has an extroverted personality. For the probability values of *p* > 0.5 and *p* < 0.5, the dogs were classified as extroverts or introverts, respectively. Thus, based on the questionnaire, the model classified the dogs into appropriate thematic groups (Figure 3).

The presented model exhibited 100% accuracy in the classification of the dogs with both extroverted and introverted temperaments. Certainly, it should be borne in mind that these were post hoc classifications, as the calculation of parameters targeted the minimization of the probability of the observed data. Therefore, the current model used for classification of new observations in the future may have a slightly lower accuracy (Table 4).

## 4. Discussion

For many years, scientists have been trying to match known human and animal personality models to animals [15]. The scientific literature distinguishes four main methods for the assessment of dog behavior, i.e., test batteries, individual assessments of dogs using a questionnaire, observations conducted under natural conditions, and expert assessments of individual breeds [16]. Other approaches for personality assessment include coding and evaluation methods. Coding involves the observation and interpretation of the behavior of the animal in a specific situation, and estimation is based on subjective attribution of a given trait [17], similar to this work.

Surveys involving owners or handlers of animals for the assessment of the temperament or behavior of said animals were introduced in the last century and are still widely used and accepted in animal personality studies [18]. Hsu and Serpell [14], Jones and Gosling [1], Diederich and Giffroy [16], and Taylor and Mills [19] reviewed the majority of the available literature reports on temperament testing, including a meta-analysis of results and methods.

The division into extroverted and introverted personality types is one of the most accurately and easily identified dimensions of human personality [20]. Analogies between human and dog personality traits have been reported by some scientists [21]. The personality types identified by the authors based on the survey results can be considered analogous to human extroversion or introversion, which is corroborated by the selection of the adjectives compiled in the table. This is a common finding in studies on many other animal species [2], including cats [22] and horses [23]. A similar division into individual personality components in chimpanzees was presented by King and Figueredo [24]. Moreover, a questionnaire-based personality assessment was performed in a group of guide dogs [14], which may emphasize its effectiveness and suitability.

Previous questionnaire-based assessments of personality or temperament have covered a very wide range of individual traits and required substantial work, e.g., over 100 questions in the study conducted by Clay et al. [11] or 152 questions in that by Hsu and Serpell [14]. Other studies, e.g., De Meester et al. [25], have focused on the assessment of only some temperamental traits, such as self-confidence, submissiveness, or timidity, without conclusively specifying the personality of the tested animals. Other investigations have consisted of asking the owner extensive questions (LAPS), which may be confusing and the answers may be puzzling; often, they do not satisfactorily determine the character of the animal [26]. In practice, the use of these methods is difficult and is sometimes even impossible due to inappropriate conditions and the owner’s weariness with the scale of difficulty. The questionnaire designed in the present study can be helpful for quick personality assessment, e.g., during a visit to a veterinary clinic or during behavioral consultation.

Although satisfactory research results have been reported, a large number of studies based on the questionnaire method require the standardization of the adjectives that describe personality and a clear division of personality based on the assessed traits. The authors of the present study hope that their method will be useful in the discrimination between extroverted and introverted personality types in dogs. Future research is planned to include the impact of sex, breed, and age on the personality of a dog. The development of tests that are not based solely on the subjective assessment of a dog’s behavior will also be investigated. The authors plan to perform short tests after subjective estimation of the temperament on the present algorithm by the caregiver, which will confirm or exclude the presence of the features marked in the questionnaire. In addition, it is planned to study a larger group of dogs and their owners in order to estimate the relationship between temperament and independent characteristics, such as gender, age, color, and breed.

## 5. Conclusions

To sum up, personality is regarded as a set of mental traits determining an animal’s reaction to a given situation. However, despite the many studies performed, there is still no reliable method for the clear assessment of dog personality. Many researchers are developing innovative methods to estimate animals’ character traits reliably. The knowledge of the personality traits of dogs is helpful in the selection of an animal for a specific role, e.g., as a guide, rescuer, or therapist. Improving the effectiveness of the adoption of shelter dogs is becoming increasingly important as well [12]. Personality tests used to assess whether a puppy will be a suitable companion for the future owner are becoming more popular [27].

To address the complexity of tests for assessing canine personality, the authors of this paper created a short questionnaire to estimate temperament as a part of canine personality. The authors of this paper were able to create this questionnaire with the help of qualified animal behaviorists and veterinarians. It is aimed at people/owners of dogs who are not familiar with animal behavior, and provides an estimate of the tendency of temperament toward extroversion or introversion based on their own observations. With the above pioneering research, any pet owner or animal caretaker at a shelter or animal hotel can estimate the temperament of a pet in a short period of time.

## Figures and Tables

**Table 1:** Authors’ questionnaire with the 24 selected temperamental traits.

Yes	Trait	No
	Wise	
	Sociable	
	Curious	
	Cheerful	
	Dominant	
	Noisy	
	Disobedient	
	Hyperactive	
	Clever	
	Impatient	
	Bold	
	Territorial	
	Stubborn	
	Loner	
	Aggressive	
	Lazy	
	Greedy	
	Alert	
	Unpredictable	
	Fearful	
	Distrustful	
	Insecure	
	Absent-minded	
	Timid	

**Table 2:** Distribution of temperament traits in both subject groups (*n*, % in the group).

Trait	Introvert	Extrovert	Total
Wise	9 50.00%	22 78.57%	31 67.39%
Sociable	1 5.56%	28 100.00%	29 63.04%
Curious	0 0.00%	22 78.57%	22 47.83%
Cheerful	1 5.56%	20 71.43%	21 45.65%
Dominant	2 11.76%	8 28.57%	10 21.74%
Noisy	5 27.78%	25 89.29%	30 65.22%
Disobedient	8 44.44%	22 78.57%	30 65.22%
Hyperactive	8 44.44%	22 78.57%	30 65.22%
Clever	2 11.11%	13 46.43%	15 32.61%
Impatient	7 38.89%	23 82.14%	30 65.22%
Bold	1 5.56%	13 46.43%	14 30.43%
Territorial	3 16.67%	23 82.14%	26 56.52%
Stubborn	16 88.89%	12 42.86%	28 60.87%
Loner	18 100.00%	0 0.00%	18 39.13%
Aggressive	6 33.33%	11 39.29%	17 36.96%
Lazy	15 83.33%	10 35.71%	25 54.35%
Greedy	17 94.44%	17 60.71%	34 73.91%
Alert	9 50.00%	11 39.29%	20 43.48%
Unpredictable	17 94.44%	5 17.86%	22 47.83%
Fearful	17 94.44%	0 0.00%	17 36.96%
Distrustful	18 100.00%	5 17.86%	23 50.00%
Insecure	16 88.89%	4 14.29%	20 43.48%
Absent-minded	11 61.11%	3 10.71%	14 30.43%
Timid	17 94.44%	0 0.00%	17 36.96%

**Table 3:** Logistic model parameters.

Parameter	$a_{0}$	$a_{1}$	$a_{2}$
Rating	−10.92	3.99	−2.39

**Table 4:** Classification of logistic model cases.

Observed	Predicted Introvert	Predicted Extrovert	Percentage
Introvert	18	0	100.00
Extrovert	0	28	100.00

## Data Availability

The data presented in this study are available on request from the corresponding authors.
